# Home-Based Health Coaching for Girls With Overweight and Obesity: A Randomized Clinical Trial

**DOI:** 10.1001/jamanetworkopen.2022.16720

**Published:** 2022-06-13

**Authors:** Richard R. Rosenkranz, Brooke J. Cull, Sara K. Rosenkranz, David A. Dzewaltowski

**Affiliations:** 1 Department of Food, Nutrition, Dietetics and Health, Kansas State University, Manhattan; 2 Department of Health Promotion, University of Nebraska Medical Center, Omaha

## Abstract

This randomized clinical trial investigated the feasibility of recruitment, delivery, and evaluation of health coaching interventions for girls.

## Introduction

Behavior-change efforts that include physical activity and healthy eating can prevent or manage complications of obesity and many noncommunicable diseases.^1^ In some regions, girls lack adequate access to health information and services, and reaching girls through evidence-based interventions in key behavioral settings has been a challenge in pediatric weight management.^2^ To investigate the potential for a definitive home-based trial, this study tested the feasibility of participant recruitment, delivery, and rigorous evaluation for an innovative girls-only health coaching intervention model.

## Methods

This randomized clinical trial was approved by Kansas State University’s institutional review board. Signed parental informed consent was obtained for all participants. The CONSORT reporting guideline was followed for reporting this trial.

In this parallel-group randomized feasibility trial conducted from 2012 to 2017, 42 girls with overweight or obesity (age range, 8-13 years) were recruited and randomly allocated (1:1 ratio) to home-based health coaching intervention conditions: 20 participants in health education (HE) and 22 participants in healthy eating and physical activity (HEPA) skills (see study protocol in Supplement 1). Full details of intervention and assessment methods are available elsewhere.^3^ Child race and ethnicity, as reported by the parent, were collected for sample description. Based on relevant behavioral theories,^4,5^ trained young adult female health coaches visited girls’ homes weekly to model healthful behaviors and deliver 60-minute coaching sessions for 12 consecutive weeks. Feasibility indicators consisted of recruitment and retention, clinical laboratory assessments, and records of intervention delivery fidelity. Clinical assessments included height, weight, waist circumference, body composition (via dual-energy X-ray absorptiometry using Lunar Prodigy, GE Healthcare), blood pressure (HEM-907XL monitor, Omron Healthcare), physical activity (Actical accelerometer, Philips), self-reported dietary intake (Children’s Dietary Questionnaire), and quality of life (Pediatric Quality of Life Inventory version 4.0). Assessments were made prerandomization at baseline, postintervention (ie, 3 months postbaseline), and at follow-up (ie, 6 months postbaseline). SPSS statistical software version 27 (IBM) was used for statistical analysis in January 2022 to derive frequencies, means, SEs, and Cohen *d*. The Figure portrays the flow of participants throughout the study.

**Figure.**
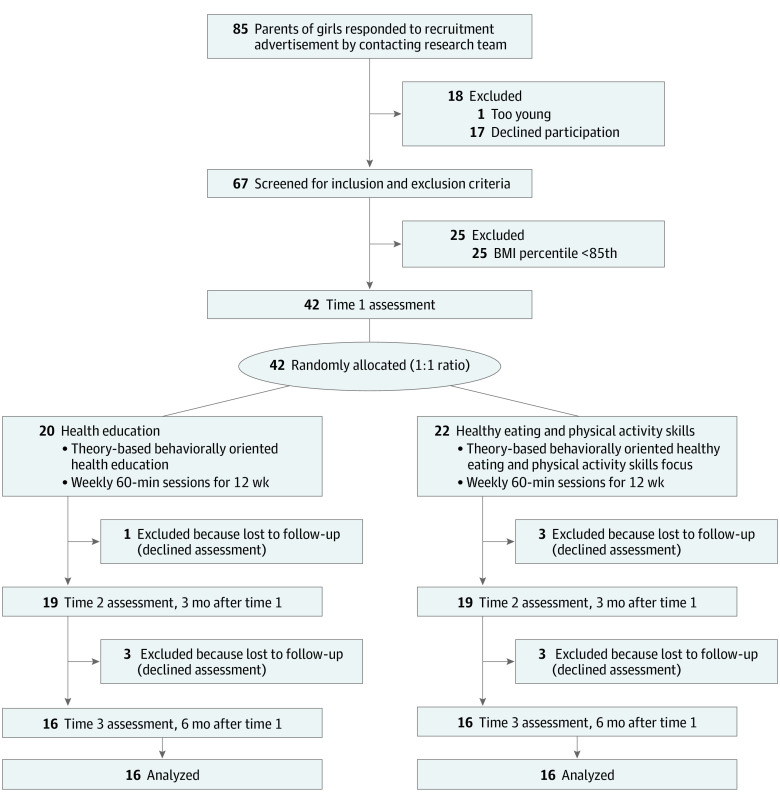
Study Flowchart BMI indicates body mass index (calculated as weight in kilograms divided by height in meters squared).

## Results

The trial was set to end after a sample size of 40 girls was achieved, requiring 4 waves of recruitment and intervention delivery (2013-2016), although a total of 42 girls were recruited. Participating girls (mean [SD] age, 10.6 [1.5] years; 2% Hispanic, 5% non-Hispanic Black or African American, 67% non-Hispanic White, 26% multiple races or ethnicities; 31% low-income households) came from mostly highly educated households (76% with maternal college degree). Girls received 97% of scheduled health coaching sessions (mean [SD], 11.7 [0.93] sessions) in their home settings, and fidelity data showed that 100% of primary and secondary session activities occurred as planned. Across 6 months of assessments, 32 participants were retained with complete data for all measures. Participants and parents reported high satisfaction with interventions; no adverse effects were reported.

The Table displays data from 3 time points by intervention condition. Effect size estimates revealed decreases in body fat percentage for both groups during the intervention period (HE: −0.48; HEPA: −0.17), with substantial attenuation of those decreases by time point 3 (HE: −0.07; HEPA: −0.04). Body mass index *z* scores also decreased during intervention as shown by effect sizes (HE: −0.35; HEPA: −0.58), and changes were maintained at time point 3 in the HEPA group (−0.78). Effect sizes for physical activity and fruit and vegetable consumption indicated small to moderate behavioral changes in the desired direction, most notably for the HEPA group. Quality of life, however, featured the largest effect sizes among all measures, with moderate to large improvements at time point 2 (HE: 0.37; HEPA: 0.65) that remained at time 3 (HE: 0.76; HEPA: 0.90).

**Table.** Biomedical, Behavioral, and Quality of Life Outcomes

Outcome	Time 1^a^		Time 2^b^		Effect size (time 2 vs 1), *d*^d^	Time 3^c^		Effect size (time 3 vs 1), *d*^d^
	Participants, No.	Mean (SE)	Participants, No.	Mean (SE)		Participants, No.	Mean (SE)	
Body fat, %								
Total	42	40.4 (1.0)	38	39.7 (1.1)	–0.33	34	40.3 (1.1)	–0.06
HE	20	41.3 (1.4)	18	40.3 (1.4)	–0.48	17	40.7 (1.1)	–0.07
HEPA skills	22	39.5 (1.4)	20	39.2 (1.7)	–0.17	17	39.9 (2.0)	–0.04
BMI *z* score								
Total	42	1.83 (0.06)	42	1.77 (0.07)	–0.44	32	1.76 (0.08)	–0.35
HE	20	1.77 (0.10)	20	1.70 (0.11)	–0.35	16	1.71 (0.11)	–0.06
HEPA skills	22	1.90 (0.08)	22	1.83 (0.09)	–0.58	16	1.82 (0.11)	–0.78
Waist circumference, cm								
Total	42	87.1 (1.3)	42	87.2 (1.4)	0.02	32	86.9 (2.2)	–0.03
HE	20	87.6 (2.1)	20	87.3 (2.0)	–0.12	16	88.5 (2.3)	0.27
HEPA skills	22	86.6 (1.8)	22	87.0 (1.9)	0.13	16	85.2 (3.7)	–0.17
BP, mm Hg								
Systolic								
Total	42	107.2 (1.6)	42	106.5 (1.4)	–0.08	32	107.2 (1.6)	–0.04
HE	20	107.3 (1.7)	20	107.9 (2.0)	0.09	16	109.2 (2.2)	0.00
HEPA skills	22	107.1 (2.7)	22	105.3 (2.0)	–0.19	16	105.2 (2.3)	–0.08
Diastolic								
Total	42	64.9 (1.3)	42	64.6 (0.9)	0.04	32	65.9 (1.0)	0.16
HE	20	63.0 (1.8)	20	63.7 (1.0)	0.09	16	66.3 (1.2)	0.28
HEPA skills	22	66.6 (1.9)	22	65.4 (1.4)	–0.15	16	65.5 (1.7)	0.03
Vigorous physical activity, min/d								
Total	41	9.2 (1.2)	38	11.7 (1.2)	0.22	NA^e^	NA	NA
HE	19	10.3 (2.2)	19	9.4 (1.3)	–0.14	NA^e^	NA	NA
HEPA skills	22	8.2 (1.2)	19	13.9 (2.1)	0.63	NA^e^	NA	NA
Steps, No./d								
Total	41	17 489 (690)	38	18 622 (737)	0.32	NA^e^	NA	NA
HE	19	17 442 (1078)	19	17 815 (921)	0.14	NA^e^	NA	NA
HEPA skills	22	17 531 (912)	19	19 428 (1144)	0.55	NA^e^	NA	NA
Fruit and vegetable intake								
d/wk								
Total	41	5.4 (0.3)	38	5.8 (0.3)	0.26	34	5.4 (0.3)	0.06
HE	19	5.1 (0.4)	19	5.4 (0.4)	0.13	16	4.6 (0.5)	–0.18
HEPA skills	22	5.7 (0.3)	19	6.2 (0.3)	0.41	18	6.1 (0.3)	0.27
Servings/d								
Total	41	3.8 (0.4)	38	4.2 (0.4)	0.19	32	3.9 (0.4)	0.06
HE	19	2.9 (0.4)	19	3.7 (0.6)	0.35	16	3.3 (0.6)	0.18
HEPA skills	22	4.5 (0.6)	19	4.6 (0.6)	0	16	4.6 (0.6)	–0.09
Quality of life								
Physical								
Total	42	25.6 (0.7)	42	27.1 (0.6)	0.39	32	27.8 (0.7)	0.63
HE	20	25.8 (1.0)	20	27.4 (0.73)	0.41	16	28.8 (0.9)	0.79
HEPA skills	22	25.4 (1.0)	22	26.8 (1.0)	0.37	16	26.8 (1.1)	0.45
Overall								
Total	42	78.3 (2.0)	38	82.4 (2.1)	0.50	32	84.5 (2.1)	0.83
HE	20	79.9 (2.6)	19	83.2 (2.7)	0.37	16	86.6 (2.6)	0.76
HEPA skills	22	76.9 (3.1)	19	81.7 (3.3)	0.65	16	82.4 (3.4)	0.90

Abbreviations: BP, blood pressure; BMI, body mass index; HE, health education; HEPA, healthy eating and physical activity; NA, not applicable.

^a^ Baseline (prior to randomization).

^b^ Postintervention.

^c^ Follow-up (3 months after intervention end).

^d^ Cohen *d* based on participants with complete data.

^e^ No physical activity data were available for time 3 owing to an information technology data storage error.

## Discussion

This randomized clinical trial evaluated the feasibility of participant recruitment, intervention delivery, and rigorous evaluation for 2 home-based health coaching interventions for pediatric weight management. Results showed that girls and parents could be recruited for participation and that trained health coaches could deliver theory-based health coaching weight-management interventions in home-based settings that were convenient and satisfactory to participants. Unknown generalizability and attrition pose limitations, but this may be less so than in other published trials.^6^ Study results suggest that proceeding to a fully powered randomized clinical trial using a standard-care control group may be warranted.

## Notes

Supplement 1.
Trial Protocol

Supplement 2.
Data Sharing Statement
