# Supplementary material for: Home-Based Health Coaching for Girls With Overweight and Obesity: A Randomized Clinical Trial
Source: JAMA Netw Open. 2022 Jun 13;5(6):e2216720. doi: 10.1001/jamanetworkopen.2022.16720 (PMC9194666; doi:10.1001/jamanetworkopen.2022.16720)
Supplement: Supplement 1. — Trial Protocol [file jamanetwopen-e2216720-s001.pdf]

FOR OFFICE USE ONLY: IRB Protocol # ____________________ Application Received: ____________
Routed: _________ Training Complete: ____________________

Committee for Research Involving Human Subjects (IRB)

Application for Approval Form
Last revised on January 2011

ADMINISTRATIVE INFORMATION:

- **Title of Project:** (if applicable, use the exact title listed in the grant/contract application)
  Wildcat Wellness Coaching Trial

- **Type of Application:**
  ☐ **New/Renewal** ☐ **Revision (to a pending new application)**
  ☒ **Modification (to an existing #__6296____ approved application)**

- **Principal Investigator:** (must be a KSU faculty member)

**Name:**	**Richard R. Rosenkranz**	**Degree/Title:**	**PhD**
**Department:**	**Human Nutrition**	**Campus Phone:**	**785 532-0152**
**Campus Address:**	**201 Justin Hall**	**Fax #:**	**785 532-3132**
**E-mail**	**ricardo@ksu.edu**		

- **Contact Name/Email/Phone for Questions/Problems with Form:** **Richard Rosenkranz/ricardo@ksu.edu/785-532-0152**

- **Does this project involve any collaborators not part of the faculty/staff at KSU?** (projects with non-KSU collaborators may require additional coordination and approvals)**:**
  ☒ **No**
  ☐ **Yes**

- **Project Classification** (Is this project part of one of the following?)**:**
  ☐ **Thesis**
  ☐ **Dissertation**
  ☒ **Faculty Research**
  ☐ **Other:**
  Note: Class Projects should use the short form application for class projects.

- **Please attach a copy of the Consent Form:**
  ☒ **Copy attached**
  ☐ **Consent form not used**

- **Funding Source:** ☒ **Internal** ☐ **External (identify source and attach a copy of the sponsor's grant application or contract as submitted to the funding agency)**
  ☐ **Copy attached** ☒ **Not applicable**

- **Based upon criteria found in 45 CFR 46 – and the overview of projects that may qualify for exemption explained at** http://www.hhs.gov/ohrp/policy/checklists/decisioncharts.html **, I believe that my project using human subjects should be determined by the IRB to be exempt from IRB review:**
  ☒ **No**
  ☐ **Yes (If yes, please complete application including Section XII. C. 'Exempt Projects'; remember that only the IRB has the authority to determine that a project is exempt from IRB review)**

If you have questions, please call the University Research Compliance Office (URCO) at 532-3224, or comply@ksu.edu

Human Subjects Research Protocol Application Form

The KSU IRB is required by law to ensure that all research involving human subjects is adequately reviewed for specific information and is approved prior to inception of any proposed activity. Consequently, it is important that you answer all questions accurately. If you need help or have questions about how to complete this application, please call the Research Compliance Office at 532-3224, or e-mail us at **comply@ksu.edu**.

Please provide the requested information in the shaded text boxes. The shaded text boxes are designed to accommodate responses within the body of the application. As you type your answers, the text boxes will expand as needed. After completion, print the form and send the original and one photocopy to the Institutional Review Board, Room 203, Fairchild Hall.

**Principal Investigator:**	**Richard R. Rosenkranz, PhD**
**Project Title:**	**Wildcat Wellness Coaching Trial**
**Date:**	**06/19/2012**

MODIFICATION

Is this a modification of an approved protocol? ☒ **Yes** ☐ **No If yes, please comply with the following:**

If you are requesting a modification or a change to an IRB approved protocol, please provide a concise description of all of the changes that you are proposing in the following block. Additionally, please highlight or bold the proposed changes in the body of the protocol where appropriate, so that it is clearly discernable to the IRB reviewers what and where the proposed changes are. This will greatly help the committee and facilitate the review.

The only change being made to the approved protocol is the addition of a non-invasive assessment for markers of inflammation and oxidative stress using a passive drool unstimulated whole (mixed) saliva collection technique at baseline, intervention end (12-weeks), and follow-up (24-weeks).

NON-TECHNICAL SYNOPSIS (brief narrative description of proposal easily understood by nonscientists):

This project involves testing the feasibility and evaluating the comparative effectiveness of two home-based coaching intervention programs through a randomized controlled trial study design. We are examining the impact of two versions of a one-hour per week coaching intervention, delivered to overweight girls in their homes by trained K-State undergraduate "coaches." Beyond determining feasibility of this type of home-based intervention, the main outcomes include changes to body composition, physical activity level, dietary habits, and quality of life.

I. BACKGROUND (concise narrative review of the literature and basis for the study):

Obesity is associated with increased chronic disease risk, and therefore poses a major public health problem (Lobstein et al., 2004). In 2011, the Centers for Disease Control and Prevention estimated that obesity affects about 12.5 million children and teens, or 17% of the US population. This is a marked increase from the ~5% rate of obesity found in this population in the late 1960s. Barlow (2007) points out that the complexity of obesity prevention lies less in the identification of target health behaviors, and much more in a process of influencing families to change behaviors when habits, culture, and environment promote less physical activity and more energy intake. Obesity prevention interventions may not be effective or sustainable without impacting home environments (Rosenkranz & Dzewaltowski, 2008). Conwell et al. (2010) suggest that home-based programs may offer significant advantages over center-based programs by offering better accessibility and convenience. Health coaching has shown promise for improving health behaviors related to chronic disease (Lawn & Schoo, 2010), but no published study has used a health coaching childhood obesity secondary prevention model in the home environment.

II. PROJECT/STUDY DESCRIPTION (please provide a concise narrative description of the proposed activity in terms that will allow the IRB or other interested parties to clearly understand what it is that you propose to do that involves human subjects. This description must be in enough detail so that IRB members can make an informed decision about proposal).

Forty female children (aged 8-13 years) will be recruited through posted flyers and word of mouth in the Manhattan, KS area. After laboratory assessment, participants will be randomly assigned to either health education (20 girls) or skills coaching (20 girls) groups. Undergraduate research assistants will undergo training to deliver interventions, each consisting of home coaching visits once

per week for 12 weeks. The skills intervention is designed to help children set goals and self-monitor healthful eating and physical activity; teach kitchen skills for fruit and vegetable snack preparation; teach children enjoyable physical activities to do at home (e.g., dancing); and provide social support for physical activity and healthful eating. The health education group will involve weekly 1 hour sessions in the home for 12 weeks, with a focus on health education and fun recreational activities. Assessments will be completed at baseline, intervention end (12 weeks), and follow-up (24 weeks). Biomedical measurements to be obtained include body composition (DEXA, tetrapolar bioimpedance, body mass index, waist circumference) blood pressure (automated sphygmomanometer), pulmonary function tests (forced expiratory flow in 1-sec, forced vital capacity, forced expiratory flow at 25-75% of vital capacity), unstimulated whole (mixed) saliva passive drool to detect markers of inflammation, and physical activity levels (7-day accelerometry). Psychosocial measurements include fruit and vegetable consumption (Child Dietary Questionnaire), self efficacy, enjoyment, and quality of life (Peds QL). Inclusion criteria: female, aged 8-13 years, body mass index at or above the sex-specific and age-adjusted 85th percentile, parental consent obtained, within 40-minute drive, and available for 12 coaching visits and lab assessments.

III. <u>OBJECTIVE</u> (briefly state the objective of the research – what you hope to learn from the study)**:**

The primary aim of this trial is to determine whether the home-based health coaching delivery model is feasible as an obesity prevention intervention strategy in the community setting. The secondary objective is to determine the comparative effectiveness of the skills coaching intervention, relative to the health education coaching intervention, in preventing increases in: body fat percentage, body mass index percentile, waist circumference, airway dysfunction, systolic and diastolic blood pressure, and inflammation. The tertiary objective is to determine comparative effectiveness on increases in: quality of life, weekly minutes of moderate-to-vigorous physical activity, physical activity self-efficacy and enjoyment, daily fruit and vegetable consumption, and self-efficacy and enjoyment for fruit and vegetable consumption. This study's findings will provide crucial information to inform further strategies for research and public health practice aimed at health protion, and prevention and treatment of obesity.

Expected findings:

We expect that we will be successful in recruiting and retaining participating families, training research assistants to collect data and deliver the intervention components, and that both the health education coaching and skills coaching conditions will be well received and appreciated by participating families.

We expect that the skills coaching intervention will be more effective than the health education coaching condition in preventing increases in blood pressure, airway dysfunction and adiposity (as measured by BMI, waist circumference, BIA, and DEXA).

We expect that both conditions will show improvements to pediatric quality of life measures, but that the skills coaching intervention will be more effective than support coaching condition in increasing physical activity, physical activity enjoyment and self efficacy, fruit and vegetable consumption, and fruit and vegetable enjoyment and self-efficacy.

IV. <u>DESIGN AND PROCEDURES</u> (succinctly outline formal plan for study)**:**

A. Location of study: **Laboratory assessments will take place in 127 Justin Hall, Kansas State University (Manhattan, KS). Coaching intervention sessions will take place in homes of study participants (around Manhattan, KS area);**

B. Variables to be studied: **Body Composition: Girls' body weight, height, and waist circumference will be assessed (in t-shirt and shorts) using a digital scale, height rod, and measurement tape. Fat and lean tissue will be assessed with DEXA. BIA will be used to determine total body water.**

Physical activity level: Girls will be asked to wear a small pedometer-like device (attached with a comfortable band at the ankle) to measure physical activity over a week-long time period. During the weekly in-home coaching sessions, research assistants will ask girls to wear a small pedometer-like device to measure physical activity during the 1-hour session.

Pulmonary/Lung Function Test (PFT):

	**The instrument for this assessment is a portable handheld spirometer, All participants will undergo standard pulmonary function tests (PFTs) by blowing into a spirometer to screen for pulmonary health.**<br><br>**Blood pressure:**<br>**Girls will be fitted with an automated blood pressure cuff to determine systolic and diastolic blood pressure**<br><br>**Inflammation:**<br>**Girls will be asked to perform an unstimulated whole (mixed) passive drool collection of saliva for multiple analytes including: IL-1beta, IL-6, CRP, TNF-alpha, and cortisol. This collection will require only one 2 ml cryovial per timepoint. The girls will be asked to allow the saliva to pool in their mouths and then will be asked to drool into a straw connected to the cryovial.**<br><br>**Questionnaires:**<br>**A parent/caregiver of each participant will complete questionnaires on basic family demographics, fruit & vegetable intake, physical activity, and participant's quality of life. Parent/caregivers and children will complete a dietary questionnaire together. Children will complete questionnaires on quality of life, self-efficacy and enjoyment related to fruits & vegetables and physical activity.**
C. Data collection methods: (surveys, instruments, etc – **PLEASE ATTACH**)	**Body composition: Dual energy X-ray absorptiometry (DEXA), tetrapolar bioelectrical impedance (BIA), height (stadiometer), weight (digital scale), waist circumference (myotape).**<br>**Physical activity: ActiCal accelerometer, Yamax pedometer, questionnaire.**<br>**Dietary intake: Child Dietary Questionnaire, parental questionnaire.**<br>**Quality of life: Peds QL, parent and child versions.**<br>**Blood pressure: Automated sphygmometer.**<br>**Inflammation: passive drool unstimulated whole (mixed) saliva.**<br>**Pulmonary Function: portable handheld spirometer.**<br>**Physical activity self-efficacy and enjoyment: questionnaire.**<br>**Fruit & vegetable self-efficacy and enjoyment: questionnaire.**
D. List any factors that might lead to a subject dropping out or withdrawing from a study. These might include, but are not limited to emotional or physical stress, pain, inconvenience, etc.:	**Participants could potentially withdraw if study activities are perceived as being tedious, unenjoyable, uncomfortable, inconvenient, or if the study activities conflict with unforeseen events.**
E. List all biological samples taken: (if any)	**None.**
F. Debriefing procedures for participants:	**After the final testing trial, the investigators will explain the procedures and tests that were performed and discuss what analyses will be performed. Additionally, the investigator will provide parent/caregivers (at their discretion) information obtained during this study as it becomes available. Parent/caregivers will also receive a summary of the group results once all the results have been obtained and analyzed.**

V. RESEARCH SUBJECTS:

A. Source: **Females ages 8-13 years from the Manhattan, KS area**

B. Number: **A total of 40 (20 for each coaching condition)**

C. Characteristics: (list any unique qualifiers desirable for research subject participation) **Inclusion criteria: Being a female aged 8 to 13 years with consenting parent or guardian. Having a BMI at or above the 85th percentile of gender-specific, age-adjusted growth charts. Family willing to participate in home-based behavioral intervention.**
Exclusion criteria:
Having developmental delay or psychiatric problems. Having any illness, injury, condition, or disease that would prevent participation in moderate-to-vigorous physical activity. Not living within 40 miles of Kansas State University campus in Manhattan, KS. Taking weight-altering medications, or participating in any other weight control program.

D. Recruitment procedures: (Explain how do you plan to recruit your subjects? Attach any fliers, posters, etc. used in recruitment. If you plan to use any inducements, ie. cash, gifts, prizes, etc., please list them here.) **Flyers posted, word of mouth, and classified advertisements in the Manhattan, KS area.**
Gift card ($20) will be given upon completion of the study, but not used for recruitment purposes

VI. RISK – PROTECTION – BENEFITS: The answers for the three questions below are central to human subjects research. You must demonstrate a reasonable balance between anticipated risks to research participants, protection strategies, and anticipated benefits to participants or others.

A. **Risks for Subjects:** (Identify any reasonably foreseeable physical, psychological, or social risks for participants. State that there are "no known risks" if appropriate.)
There is potential for embarrassment or sensitivity regarding measurement of weight, waist circumference, and reporting of dietary and physical activity habits. There is potential for minor musculoskeletal injury from increased physical activity (e.g., dancing).

B. **Minimizing Risk:** (Describe specific measures used to minimize or protect subjects from anticipated risks.)

In order to avoid embarrassment of weight and waist circumference measurements, these measurements will be taken in a private location. Reported dietary and physical activity data will be kept confidential.

The majority of our procedures have previously been approved by KSU IRB (including DEXA). DEXA carries minimal xray exposure (see table below) equivalent of 6-8 hrs of sunlight)(mSv = millisievert) (Sources: American college of Radiology, Radiological Society of North America, American Association of Physical Medicine, The New England Journal of Medicine; University of California, San Francisco, Cancer Center). To minimize exposure, the DXA procedure will only be performed once and BIA will be utilized as our pre-post body composition assessment.

Radiation Amount
CT scan, full body 10-12 mSv
CT scan, chest or pelvis 4-8 mSv
Natural background radiation (from sunlight, radon gas, etc.) from living in high-altitude cities (e.g., Denver, Salt Lake City) 6 mSv (per year)
Natural background radiation from living at sea level 3 mSv (per year)
Mammogram 1-2 mSv
High-mileage frequent flying (100,000-450,000 miles per year) 1-6.7 mSv
X-ray of chest (or ankle to look for broken bones) 0.1-0.6 mSv
DEXA (bone-density) scan 0.01-0.05 mSv (body comp scan is about 1/10th of this amount)
Dental X-ray (bitewing) 0.02 mSv
Single airplane flight, coast-to-coast 0.01-0.03 mSv

C. **Benefits:** (Describe any reasonably expected benefits for research participants, a class of participants, or to society as a whole.)

Participants will likely benefit from the supportive interaction with the intervention coach. At the conclusion of the project, parent/caregivers will obtain their daughter's laboratory measurement information related to the study. Parent/caregivers may benefit from their daughter's information on body composition, blood pressure, pulmonary function, physical activity, fruit & vegetable consumption, and quality of life.

In your opinion, does the research involve **more than minimal risk** to subjects? ("Minimal risk" means that "the risks of harm anticipated in the proposed research are not greater, considering probability and magnitude, than those ordinarily encountered in daily life or during the performance of routine physical or psychological examinations or tests.")

☐ **Yes** ☒ **No**

VII. **CONFIDENTIALITY:** Confidentiality is the formal treatment of information that an individual has disclosed to you in a relationship of trust and with the expectation that it will not be divulged to others without permission in ways that are inconsistent with the understanding of the original disclosure. Consequently, it is your responsibility to protect information that you gather from human research subjects in a way that is consistent with your agreement with the volunteer and with their expectations. If possible, it is best if research subjects' identity and linkage to information or data remains unknown.

Explain how you are going to protect confidentiality of research subjects and/or data or records. Include plans for maintaining records after completion.

Subjects data will not be identified by name, but by coded ID number. No identifiable markers will be used in published reports from the study.

VIII. INFORMED CONSENT: Informed consent is a critical component of human subjects research – it is your responsibility to make sure that any potential subject knows exactly what the project that you are planning is about, and what his/her potential role is. (There may be projects where some forms of "deception" of the subject is necessary for the execution of the study, but it must be carefully justified to and approved by the IRB). A schematic for determining when a

waiver or alteration of informed consent may be considered by the IRB is found at

http://www.hhs.gov/ohrp/policy/consentckls.html

Even if your proposed activity does qualify for a waiver of informed consent, you must still provide potential participants with basic information that informs them of their rights as subjects, i.e. explanation that the project is research and the purpose of the research, length of study, study procedures, debriefing issues to include anticipated benefits, study and administrative contact information, confidentiality strategy, and the fact that participation is entirely voluntary and can be terminated at any time without penalty, etc. Even if your potential subjects are completely anonymous, you are obliged to provide them (and the IRB) with basic information about your project. See informed consent example on the URCO website. It is a federal requirement to maintain informed consent forms for 3 years after the study completion.

Yes	No	**Answer the following questions about the informed consent procedures.**
☒	☐	A. Are you using a written informed consent form? If "yes," include a copy with this application. If "no" see b.
☐	☒	B. In accordance with guidance in 45 CFR 46, I am requesting a waiver or alteration of informed consent elements (See Section VII above). If "yes," provide a basis and/or justification for your request.
☒	☐	C. Are you using the online Consent Form Template provided by the URCO? If "no," does your Informed Consent document has all the minimum required elements of informed consent found in the Consent Form Template? (Please explain)
☐	☒	D. Are your research subjects anonymous? If they are anonymous, you will not have access to any information that will allow you to determine the identity of the research subjects in your study, or to link research data to a specific individual in any way. Anonymity is a powerful protection for potential research subjects. (An anonymous subject is one whose identity is unknown even to the researcher, or the data or information collected cannot be linked in any way to a specific person).
☒	☐	E. Are subjects debriefed about the purposes, consequences, and benefits of the research? Debriefing refers to a mechanism for informing the research subjects of the results or conclusions, after the data is collected and analyzed, and the study is over. (If "no" explain why.) Attach copy of debriefing statement to be utilized.

***It is a requirement that you maintain all signed copies of informed consent documents for at least 3 years following the completion of your study. These documents must be available for examination and review by federal compliance officials.**

IX. PROJECT INFORMATION: (If you answer yes to any of the questions below, you should explain them in one of the paragraphs above)

Yes	No	**Does the project involve any of the following?**
☐	☒	a. Deception of subjects
☐	☒	b. Shock or other forms of punishment
☐	☒	c. Sexually explicit materials or questions about sexual orientation, sexual experience or sexual abuse
☐	☒	d. Handling of money or other valuable commodities
☐	☒	e. Extraction or use of blood, other bodily fluids, or tissues
☐	☒	f. Questions about any kind of illegal or illicit activity
☐	☒	g. Purposeful creation of anxiety
☐	☒	h. Any procedure that might be viewed as invasion of privacy
☒	☐	i. Physical exercise or stress
☐	☒	j. Administration of substances (food, drugs, etc.) to subjects
☐	☒	k. Any procedure that might place subjects at risk
☐	☒	l. Any form of potential abuse; i.e., psychological, physical, sexual
☒	☐	m. Is there potential for the data from this project to be published in a journal, presented at a conference, etc?

☒	☐	n.	Use of surveys or questionnaires for data collection<br>**IF YES, PLEASE ATTACH!!**

X. <u>SUBJECT INFORMATION</u>: (If you answer yes to any of the questions below, you should explain them in one of the paragraphs above)

Yes	No		**Does the research involve subjects from any of the following categories?**
☒	☐	a.	Under 18 years of age (these subjects require parental or guardian consent)
☐	☒	b.	Over 65 years of age
☐	☒	c.	Physically or mentally disabled
☐	☒	d.	Economically or educationally disadvantaged
☒	☐	e.	Unable to provide their own legal informed consent
☐	☒	f.	Pregnant females as target population
☐	☒	g.	Victims
☐	☒	h.	Subjects in institutions (e.g., prisons, nursing homes, halfway houses)
☐	☒	i.	Are research subjects in this activity students recruited from university classes or volunteer pools? If so, do you have a reasonable alternative(s) to participation as a research subject in your project, i.e., another activity such as writing or reading that would serve to protect students from unfair pressure or coercion to participate in this project? If you answered this question "Yes," explain any <u>alternatives options</u> for class credit for potential human subject volunteers in your study. (It is also important to remember that: Students must be free to choose **not** to participate in research that they have signed up for **at any time** without penalty. Communication of their decision can be conveyed in any manner, to include **simply not showing up** for the research.)
☐	☒	j.	Are research subjects **audio** taped? If yes, how do you plan to protect the recorded information and mitigate any additional risks?
☐	☒	k.	Are research subjects' images being recorded (video taped, photographed)? If yes, how do you plan to protect the recorded information and mitigate any additional risks?

XI. **<u>CONFLICT OF INTEREST</u>:** Concerns have been growing that financial interests in research may threaten the safety and rights of human research subjects. Financial interests are not in them selves prohibited and may well be appropriate and legitimate. Not all financial interests cause Conflict of Interest (COI) or harm to human subjects. However, to the extent that financial interests may affect the welfare of human subjects in research, IRB's, institutions, and investigators must consider what actions regarding financial interests may be necessary to protect human subjects. Please answer the following questions:

Yes	No		
☐	☒	a.	Do you or the institution have any proprietary interest in a potential product of this research, including patents, trademarks, copyrights, or licensing agreements?
☐	☒	b.	Do you have an equity interest in the research sponsor (publicly held or a non-publicly held company)?
☐	☒	c.	Do you receive significant payments of other sorts, eg., grants, equipment, retainers for consultation and/or honoraria from the sponsor of this research?
☐	☒	d.	Do you receive payment per participant or incentive payments?
☐	☒	e.	If you answered yes on any of the above questions, please provide adequate explanatory information so the IRB can assess any potential COI indicated above.

XII. PROJECT COLLABORATORS:

A. **KSU Collaborators – list anyone affiliated with KSU who is collecting or analyzing data:** (list all collaborators on the project, including co-principal investigators, undergraduate and graduate students)

Name:	Department:	Campus Phone:	Campus Email:
**Sara Rosenkranz**	**Human Nutrition**	**785-532-1465**	**SaraRose@ksu.edu**
**David A Dzewaltowski**	**Kinesiology**	**785-532-7795**	**dadx@ksu.edu**
**Hugh Rosett**	**Human Nutrition**	**785-532-0170**	**rosett@ksu.edu**
**Brooke Cull**	**Human Nutrition**	**785-532-0170**	**brooke1@ksu.edu**

B. **Non-KSU Collaborators:** (List all collaborators on your human subjects research project not affiliated with KSU in the spaces below. KSU has negotiated an Assurance with the Office for Human Research Protections (OHRP), the federal office responsible for oversight of research involving human subjects. When research involving human subjects includes collaborators who are not employees or agents of KSU the activities of those unaffiliated individuals may be covered under the KSU Assurance only in accordance with a formal, written agreement of commitment to relevant human subject protection policies and IRB oversight. The Unaffiliated Investigators Agreement can be found and downloaded at http://www.k-state.edu/research/comply/irb/forms/Unaffiliated%20Investigator%20Agreement.doc

C.

The URCO must have a copy of the Unaffiliated Investigator Agreement on file for each non-KSU collaborator who is not covered by their own IRB and assurance with OHRP. Consequently, it is critical that you identify non-KSU collaborators, and initiate any coordination and/or approval process early, to minimize delays caused by administrative requirements.)

Name:	Organization:	Phone:	Institutional Email:

Does your non-KSU collaborator's organization have an Assurance with OHRP? (for Federalwide Assurance and Multiple Project Assurance (MPA) listings of other institutions, please reference the OHRP website under Assurance Information at: http://ohrp.cit.nih.gov/search).

☐ **No**
☐ **Yes** **If yes, Collaborator's FWA or MPA #** ______

Is your non-KSU collaborator's IRB reviewing this proposal?

☐ **No**
☐ **Yes** **If yes, IRB approval #** ______

C. **Exempt Projects:** 45 CFR 46 identifies six categories of research involving human subjects that may be exempt from IRB review. The categories for exemption are listed here: http://www.hhs.gov/ohrp/policy/checklists/decisioncharts.html. If you believe that your project qualifies for exemption, please indicate which exemption category applies (1-6). Please remember that only the IRB can make the final determination whether a project is exempt from IRB review, or not.

Exemption Category: ______

XIII. CLINICAL TRIAL ☐Yes ☒No

(If so, please give product.)

Export Controls Training:
-The Provost has mandated that all KSU faculty/staff with a full-time appointment participate in the Export Control Program.
-If you are not in our database as having completed the Export Control training, this proposal will not be approved until your participation is verified.
-To complete the Export Control training, follow the instructions below:
Click on:

http://www.k-state.edu/research/comply/ecp/index.htm

1. After signing into K-State Online, you will be taken to the Export Control Homepage
2. Read the directions and click on the video link to begin the program
3. Make sure you enter your name / email when prompted so that participation is verified

If you click on the link and are not taken to K-State Online, this means that you have already completed the Export Control training and have been removed from the roster. If this is the case, no further action is required.

-Can't recall if you have completed this training? Contact the URCO at 785-532-3224 or comply@ksu.edu and we will be happy to look it up for you.

Post Approval Monitoring: The URCO has a Post-Approval Monitoring (PAM) program to help assure that activities are performed in accordance with provisions or procedures approved by the IRB. Accordingly, the URCO staff will arrange a PAM visit as appropriate; to assess compliance with approved activities.

If you have questions, please call the University Research Compliance Office (URCO) at 532-3224, or comply@ksu.edu

INVESTIGATOR ASSURANCE FOR RESEARCH INVOLVING HUMAN SUBJECTS

(Print this page separately because it requires a signature by the PI.)

P.I. Name: **Richard R. Rosenkranz, PhD**

Title of Project: **Wildcat Wellness Coaching Trial**

XIV. ASSURANCES: As the Principal Investigator on this protocol, I provide assurances for the following:

A. **Research Involving Human Subjects: This project will be performed in the manner described in this proposal, and in accordance with the Federalwide Assurance FWA00000865 approved for Kansas State University available at http://ohrp.osophs.dhhs.gov/polasur.htm#FWA, applicable laws, regulations, and guidelines. Any proposed deviation or modification from the procedures detailed herein must be submitted to the IRB, and be approved by the Committee for Research Involving Human Subjects (IRB) prior to implementation.**

B. **Training: I assure that all personnel working with human subjects described in this protocol are technically competent for the role described for them, and have completed the required IRB training modules found on the URCO website at: http://www.k-state.edu/research/comply/irb/training/index.htm. I understand that no proposals will receive final IRB approval until the URCO has documentation of completion of training by all appropriate personnel.**

C. **Extramural Funding: If funded by an extramural source, I assure that this application accurately reflects all procedures involving human subjects as described in the grant/contract proposal to the funding agency. I also assure that I will notify the IRB/URCO, the KSU PreAward Services, and the funding/contract entity if there are modifications or changes made to the protocol after the initial submission to the funding agency.**

D. **Study Duration: I understand that it is the responsibility of the Committee for Research Involving Human Subjects (IRB) to perform continuing reviews of human subjects research as necessary. I also understand that as continuing reviews are conducted, it is my responsibility to provide timely and accurate review or update information when requested, to include notification of the IRB/URCO when my study is changed or completed.**

E. **Conflict of Interest: I assure that I have accurately described (in this application) any potential Conflict of Interest that my collaborators, the University, or I may have in association with this proposed research activity.**

F. **Adverse Event Reporting: I assure that I will promptly report to the IRB / URCO any unanticipated problems involving risks to subjects or others that involve the protocol as approved. Unanticipated or Adverse Event Form is located on the URCO website at: http://www.k-state.edu/research/comply/irb/forms/index.htm. In the case of a serious event, the Unanticipated or Adverse Events Form may follow a phone call or email contact with the URCO.**

G. **Accuracy: I assure that the information herein provided to the Committee for Human Subjects Research is to the best of my knowledge complete and accurate.**

____________________________________ ________________

(Principal Investigator Signature) **(date)**
