# Supplementary material for: Home-Based Health Coaching for Girls With Overweight and Obesity: A Randomized Clinical Trial
Source: JAMA Netw Open. 2022 Jun 13;5(6):e2216720. doi: 10.1001/jamanetworkopen.2022.16720 (PMC9194666; doi:10.1001/jamanetworkopen.2022.16720)
Supplement: Supplement 2. — Data Sharing Statement [file jamanetwopen-e2216720-s002.pdf]

Data Sharing Statement

Rosenkranz R, Cull BJ, Rosenkranz SK, Dzewaltowski DA. Home-based health coaching for girls with overweight and obesity. *JAMA Netw Open*. 2022;5(6):e2216720. doi:10.1001/jamanetworkopen.2022.16720

Data
Data available: Yes
Data types: Deidentified participant data
How to access data: Email ricardo@ksu.edu
When available: With publication

Supporting Documents
Document types: Informed consent form
How to access documents: email ricardo@ksu.edu
When available: With publication

Additional Information
Who can access the data: anyone requesting the data
Types of analyses: for any purpose
Mechanisms of data availability: without investigator support
